# Hydrogen Bond and Other Lewis Acid–Lewis Base Interactions as Preliminary Stages of Chemical Reactions

**DOI:** 10.3390/molecules25204668

**Published:** 2020-10-13

**Authors:** Sławomir J. Grabowski

**Affiliations:** 1 Polimero eta Material Aurreratuak: Fisika, Kimika eta Teknologia, Kimika Fakultatea, Euskal Herriko Unibertsitatea UPV/EHU & Donostia International Physics Center (DIPC) PK 1072, 20080 Donostia, Euskadi, Spain; s.grabowski@ikerbasque.org; Tel.: +34-943-018-187; 2 IKERBASQUE, Basque Foundation for Science, 48011 Bilbao, Spain

**Keywords:** Lewis acid–Lewis base interactions, hydrogen bond, tetrel bond, pnicogen bond, triel bond, electron charge shifts

## Abstract

Various Lewis acid–Lewis base interactions are discussed as initiating chemical reactions and processes. For example, the hydrogen bond is often a preliminary stage of the proton transfer process or the tetrel and pnicogen bonds lead sometimes to the S_N_2 reactions. There are numerous characteristics of interactions being first stages of reactions; one can observe a meaningful electron charge transfer from the Lewis base unit to the Lewis acid; such interactions possess at least partly covalent character, one can mention other features. The results of different methods and approaches that are applied in numerous studies to describe the character of interactions are presented here. These are, for example, the results of the Quantum Theory of Atoms in Molecules, of the decomposition of the energy of interaction or of the structure-correlation method.

## 1. Introduction

It is well known that the hydrogen bond plays a crucial role in numerous chemical, physical and biological processes [1,2]. However, other interactions are also important in various processes, particularly biochemical ones [3,4]. It is worth mentioning that such terms as interaction and reaction are even used interchangeably sometimes since interactions often lead to corresponding reactions or at least they are initiative steps of chemical reactions. The aim of this study is to display dependencies between the latter phenomena. The interrelations between interactions and reactions as well as between them and other phenomena and processes, or reasons for the lack of such relations, were discussed in numerous studies, even in very early ones. For example, Lewis has described that “at the recent conference of the Faraday Society (July, 1923) all of those who participated seemed agreed that the average organic molecule is very little polarized, but there were some who believed that polarisation and indeed ionisation precede every reaction” [5].

More recent studies indicate an important role of electron charge shifts (related to the polarisation) in processes corresponding to interactions and then to chemical reactions. For example, Rauk has stated that “all reactions of organic compounds are treated within the framework of generalized Lewis acid - Lewis base theory, their reactivity being governed by the characteristics of the frontier orbitals of the two reactants. All compounds have occupied molecular orbitals and so can donate electrons, that is, act as bases in the Lewis sense. All compounds have empty molecular orbitals and so can accept electrons, that is, act as acids in the Lewis sense” [6].

It was also indicated that the term “noncovalent interactions” is not a proper one since it concerns hydrogen bond and halogen bond as well as other interactions that often possess characteristics of covalent bonds [7]. The covalent character is often related to the occurrence of polarization and charge transfer processes [8,9]. On the other hand, for very weak interactions ruled mainly by dispersion forces, the electron charge shifts are negligible, if there are any, also electrostatic interactions are not important there. This is why the term Lewis acid–Lewis base interactions seems to be the proper one since it is related to those interactions where the electron charge shifts occur between Lewis acid and Lewis base units. It seems also that this term excludes very weak interactions, as those that occur between methane molecules, for example, where dispersion forces are the most important ones and where it is difficult to indicate the Lewis acid and Lewis base centres [7].

Kaplan has concluded in his monograph that “intermolecular interactions are involved in the formation of complicated chemical complexes, such as charge-transfer and hydrogen-bond complexes. Study of the mechanism of elementary chemical reactions is impossible without knowledge of the exchange processes between the translational and electron-vibration energies, which depend on the interaction of particles under collisions. Knowledge of the potential surface, characterizing the mutual trajectories of the reactants, is necessary to obtain the rates of chemical reactions” [10].

This is why the aim of this review is to point out relationships between interactions and reactions since the former phenomena may lead to the latter ones thus they initiate numerous structural changes and electron charge redistributions in species being in contact. This is of particular interest, which characteristics possess interactions that lead to chemical reactions. It was discussed in one of recent reviews that the stronger electrostatic interactions lead to the stronger Pauli repulsion that implies the greater electron charge shifts, mainly from the Lewis base unit to the Lewis acid; these phenomena may lead to the chemical reaction [11]. In this review, few types of interactions are discussed and it is analysed which conditions should be fulfilled for them that they lead to chemical reactions.

## 2. The Hydrogen Bond as a Preliminary Stage of the Proton Transfer Process

It has been described in early studies that a fragment of a crystal structure may be treated as “a frozen stage” of a considered chemical reaction. The related fragments that differ by geometry and that are taken from various crystal structures may correspond to the analysed chemical reaction since they reflect structural changes accompanying this reaction [12,13,14]. This approach is known as the structure-correlation method. It was applied to analyse different reactions such as the nucleophilic addition to a carbonyl group, nucleophilic substitution at tetrahedral coordinated atoms (S_N_1 and S_N_2 reactions); electrocyclic ring closure of polyenes and other chemical processes [12,13,14,15].

It is important that the proton transfer, PT, process related to the hydrogen bond, HB, may be also discussed in terms of the structure-correlation method. For example, PT in O-H···O hydrogen bonds was analysed since -C=O···H-O-C- fragments taken from different crystal structures were compared to reconstruct the corresponding reaction path [9,11,16]. For these analyses, the high-precision neutron diffraction geometries were taken from the Cambridge Structural Database, CSD [17,18]. The recent CSD release (CSD updates up to May 2020) was applied here to search the above-mentioned -C=O···H-O-C- fragment with the following search criteria; accurate crystal structures with e.s.d’s ≤ 0.005 Å, R ≤ 7.5%, error free structures, without disorder, no polymers and no powder diffraction results. Only neutron diffraction results were taken into account here since they are characterised by precisely determined positions of H-atoms [19] in contrast to the X-ray results, where the refinement of crystal structures is usually based on the spherical approximation of the atomic electron densities that results in the spherical symmetry of atomic scattering factors [20]. One may say that the sample of fragments described above and corresponding to different crystal structures of organic and organometallic compounds may reflect the reaction path of the following PT process; -C=O···H-O-C- ⇔ -C-O-H···O=C-. In some of structures H-atom is situated in the mid-point of the O...O distance or near to this point therefore -C=O···H^+^···O=C- fragments are also included in the sample. The search has led to a finding of 56 geometrical fragments corresponding to the above-mentioned PT process. The similar search with the same criteria for accuracy of results was performed for the similar fragments where the H-atom is replaced by the deuterium, i.e., the -C=O...D-O-C- fragments; in this case only four structures were found.

**Figure 1:**
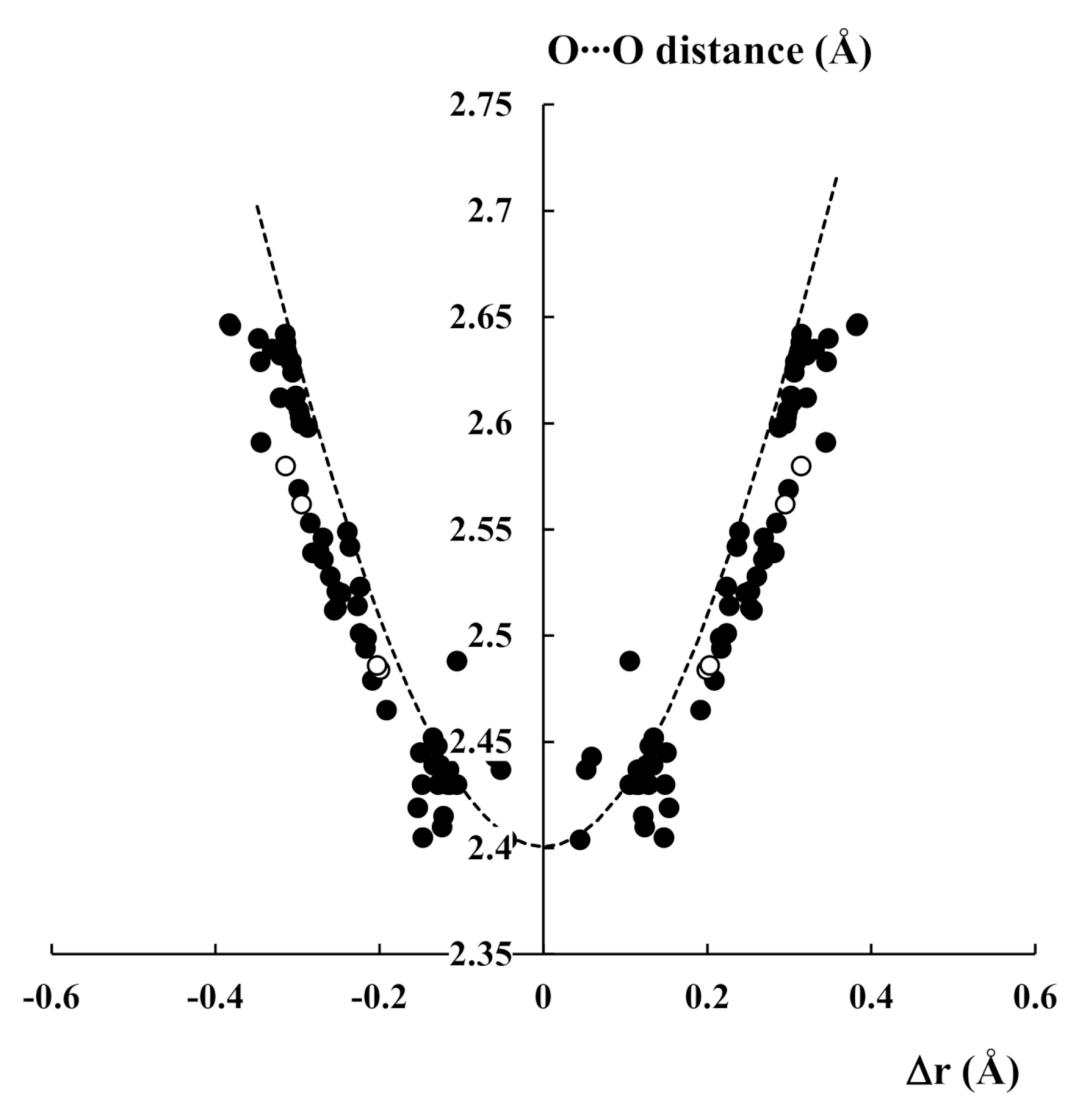
The dependence between the Δr (Å) - the displacement of the proton (or deuter) position from the O···O mid-point and the O···O distance (Å), for the O-H···O systems (black circles) and the O-D···O ones (white circles). The broken line corresponds to the bond number conservation rule (Equation (1)).

Figure 1 presents the PT reaction path based on two above described CSD searches; this is the relationship between the Δr parameter and the O···O distance. The same relationships were discussed before [9,11,16] but they were based on earlier CSD updates. The Δr parameter is the distance of the H-atom of the O-H...O bridge from the O···O mid-point. For the linear O-H(D) ···O systems the O···O distance may be expressed as the r_O-H_ + r_H…O_ sum while the Δr parameter as the (r_H…O_ − r_OH_)/2 term. The r_OH_ and r_H…O_ values correspond to the O-H bond length and the H…O distance, respectively. The points of Figure 1, which correspond to fragments of crystal structures, may be considered as positions of the proton in PT process. The results of this figure are symmetrised around Δr = 0; this symmetrisation corresponds to the equivalency of systems during PT reaction because the homonuclear O-H···O hydrogen bond is discussed here. The “points” in the middle of O···O distance may be considered as the transition state of the proton transfer reaction. These are strongly elongated O-H bonds and they are observed for the O···O distances amounting about ~2.4–2.5 Å. For long O···O distances the H-atoms are located far from their mid-points, they are situated close to one of oxygen centres rather.

The broken line of Figure 1 corresponds to the relationship expressing the bond order (number) conservation rule (Equation (1)) [12,13,21].

(1) $$\exp\left(\frac{\Delta r_{i}}{c}\right)+\exp\;\left(\frac{\Delta r_{j}}{c}\right)\;=1$$

The $\Delta r_{i}$ and $\Delta r_{j}$ terms in the above equation correspond to the (r_0_ − r_OH_) and (r_0_ − r_H∙∙∙O_) differences; r_0_ is the typical single O-H bond length not perturbed by any interaction. The bond length of water in the gas phase equal to 0.957 Å was chosen here and in other studies [9]. The exponential terms of Equation (1), exp(Δr_i_/c), may be treated as the definition of the bond order. However, other names for this term as well as for similar expressions are often applied in various studies; the bond valence [21] or the bond number [22], for example. The constant c for the O-H···O hydrogen bonds is determined from the above exponential expression assuming that for the O-H···O linear system with the H-atom located in the O···O mid-point, and the O···O distance of 2.4 Å, two equivalent H···O distances possess the bond order equal to 0.5; in other words; c = (0.957 − 1.2)/ln(0.5).

The bond order and the bond order conservation rule ideas for the O-H···O hydrogen bonds may be described in the following way. For the single O-H bond in the gas phase, the bond order is equal to unity. If this bond is involved in the hydrogen bond thus it is elongated and its bond order decreases. However, this decrease is compensated by the H···O contact. Hence the sum of bond orders (Equation (1)) of the O-H bond and the H···O contact is equal to unity. The greater is the O-H bond elongation for the stronger hydrogen bond thus the greater is the bond order of the H···O contact that is shorter accordingly; the latter is also accompanied by the decrease of the O···O distance (Figure 1). The extreme cases of very short O···O distances with the H-atom location at the O···O mid-point for very strong hydrogen bonds correspond to the transition states of the proton transfer reaction.

One can see that the broken line of Figure 1 that was derived from the bond order conservation rule is only approximately in agreement with the neutron diffraction results. Similar disagreement concerning the relationship between the O-H bond length and the H···O distance was explained by the influence of electrostatic forces that are not properly included in the bond order conservation concept [23]. Hence the corrected reference single O-H bond length of 0.925 Å was proposed to take into account the electrostatic contribution and to have better agreement between experimental results and theoretical evaluations [23]. Figure 1 contains also the O-D···O systems; it is pointed out in several studies that the deuteration of the O-H···O systems results in shortening of the O-D bond and lengthening of the O···O and D···O distances in comparison with their non-deuterated counterparts; it is known as the Ubbelohde effect [24]. Figure 1 shows that the deuterated O-D···O systems are approximately in agreement with the broken curve derived from Equation (1). One may conclude that the reaction path presented in Figure 1 shows that the hydrogen bond, especially for the strong O-H···O interactions, may be treated as the initial stage of PT process.

It is worth mentioning that similar relationships to this one presented in Figure 1 were analysed for other hydrogen bonded systems. For example, it was found that the dependence between q_1_ and q_2_ parameters for the N-H···N hydrogen bond geometries taken from experimental NMR and crystal structures´ results is in agreement with the bond order conservation rule expressed by an equation similar to that one presented above here (Equation (1)) [25]. The q_1_ and q_2_ parameters are equal to (r_H…N_ − r_N-H_)/2 and r_H…N_ + r_N-H_, respectively; r_H…N_ is the H···N distance and r_N-H_ is the N-H bond length in the N-H···N system.

In another study the proton bound water dimer, H_5_O_2_^+^ (Zundel cation) was discussed [26]. The relationships between molecular structures of this cation and the ^1^H-NMR chemical shifts were presented; low-temperature neutron diffraction results were used for these relationships. The dependence between q_1_ = (r_H…O_ − r_O-H_)/2 and q_2_ = r_H…O_ + r_O-H_ parameters that is very similar to that one presented in Figure 1 has been also discussed [26]. The chemical shifts and other NMR parameters were also analysed for the N-H···N [25,27], F-H···N [28] and F-H···F^−^ [28] hydrogen bonded systems. The bond order conservation rule [9,12] was compared there with the experimental results and with the theoretical calculations [25,27,28].

One can see that the geometries of hydrogen bonded systems represent various stages of the proton transfer reaction. It is discussed above here for different types of hydrogen bonds. As a consequence, the question arises, what are the characteristics of hydrogen bonds that may be treated as the initial stages of the PT process? It was discussed in earlier studies [9,11,29] that the strong hydrogen bonds may lead to the proton transfer reactions. It is worth recalling here effects that accompany the A-H···B hydrogen bond formation. It is the Lewis acid–Lewis base interaction thus the noticeable electron charge shift from the base unit to the acidic one occurs here [7,11]. This is connected with the n_B_ → σ_AH_^*^ orbital-orbital interaction [8], where n_B_ is the lone electron pair orbital of the B-centre while σ_AH_^*^ is the antibonding orbital of the A-H σ-bond. If we exclude from our discussion the blue shift hydrogen bonds [30] which do not lead to the PT process rather thus the hydrogen bond formation is connected with the increase of the polarization of the A-H bond, its elongation and consequently with its weakening. In extreme cases of the strong hydrogen bonds possessing covalent or at least partly covalent character [9] the PT reaction occurs. The terms resulting from the decomposition of the energy of interaction and related to the electron charge shifts are described as those expressing the covalency. The following terms occur in different decomposition schemes: charge transfer, polarization, delocalization or induction term. The name of term depends on the decomposition scheme applied. The interaction energy contributions differ by physical meaning in different schemes although they similarly express processes related to the electron charge shifts.

The covalent character may be also detected by the Quantum Theory of Atoms in Molecules (QTAIM) approach [31,32]. The value of the electron density at the H···B bond critical point (BCP), ρ_BCP_, of the order of 0.1 u and more, and the negative Laplacian of this electron density, ∇^2^ρ_BCP_, inform of the covalent character of the hydrogen bond. However, it is assumed in numerous studies that even for ∇^2^ρ_BCP_ > 0, the negative value of the total electron energy density at BCP, H_BCP_, shows the partly covalent character of interaction [9,33,34,35,36].

**Figure 2:**
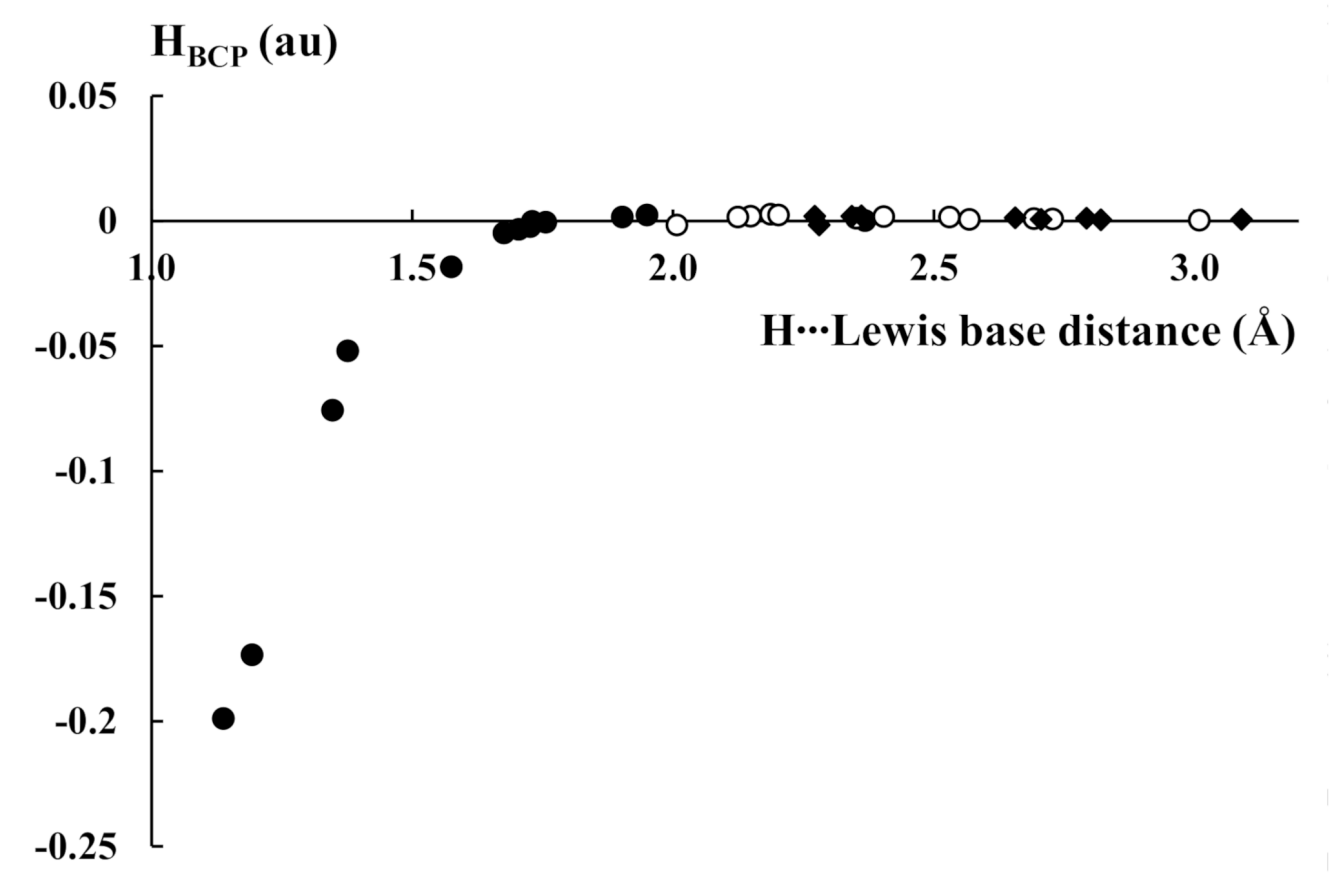
The dependence between the H···Lewis base distance (between H-atom of the proton donor and the centre of Lewis base unit, in Å) and the total electron energy density at BCP, H_BCP_ for hydrogen bonded systems. The Lewis base centre is an atom for 3c–4e A-H···B hydrogen bonds (black squares for C-H···B hydrogen bonds and black circles for other 3c–4e systems) and it is BCP of CC bond or C-atom in Lewis base unit for the A-H···π hydrogen bonds (while circles).

Figure 2 presents the relationship between the hydrogen···Lewis base distance and the H_BCP_ value at the corresponding BCP, for the sample of hydrogen bonds analysed in earlier study [37]. These results are based on the MP2/6-311++G(d,p) calculations. The following complexes linked by hydrogen bonds were discussed there. The complexes connected by charge assisted hydrogen bonds (CAHBs): (FHF)^−^, H_2_O···H_3_O^+^, H_3_O^+^···HCN, OH^−^···H_2_O, NH_3_···NH_4_^+^, C_5_H_5_^−^···HF and C_5_H_5_^−^···C_2_H_2_. The complexes with π-electrons acting as the acceptor of proton, these are two latter CAHB systems as well as the following species; C_2_H_2_···HF, C_2_H_4_···HF, C_6_H_6_···HF, (C_2_H_2_)_2_ (T-shaped dimer), C_2_H_4_···C_2_H_2_, C_6_H_6_···C_2_H_2_, C_6_H_6_···CH_4_, C_6_H_6_···CHCl_3_, C_2_H_2_···CH_4_ and C_2_H_2_···CHCl_3_. There is the sub-sample of complexes linked by the C-H···B hydrogen bonds: F_3_CH···NCCH_3_, H_3_CH···NCCH_3_, HCCH···NCCH_3_, F_3_CH···OCH_2_, H_3_CH···OCH_2_, HCCH···OCH_2_, H_3_CH···SH_2_, HCCH···SH_2_ and HCCH···S(CH_3_)_2_. The other hydrogen bonds analysed in the above-mentioned study [37] may be classified as moderate or strong ones, these are interactions in the following complexes; (C_6_H_5_COOH)_2_, (CH_3_COOH)_2_, (HCOOH)_2_, (HCONH)_2_, (HCSNH)_2_, (H_2_O)_2_ (trans-linear dimer), H_2_O···HF and H_2_CO···HF. The hydrogen bonds are divided into three groups in Figure 2, C-H···π (open circles, the C_5_H_5_^−^···HF complex with the F-H proton donating bond is also included there), C-H···B (black squares) and remaining ones, among them CAHB systems (black circles). The hydrogen···Lewis base distance is understood here in the following way: it is the H···B distance for the 3c–4e (three centre–four electron [8]) A-H···B hydrogen bonds. For the C-H···π interactions this is the distance between the H-atom of Lewis acid unit and the carbon atom or the bond critical point of CC bond of the Lewis base [37]. The latter depends on the kind of the bond path characterizing the intermolecular link [37]. Figure 2 shows that for stronger interactions characterised by shorter distances between Lewis acid-base units the covalent character is revealed that is expressed by the negative H_BCP_ values, the corresponding systems may be treated as the potentially preliminary stages of PT processes. The weaker hydrogen bonds are characterised by longer distances between these units, these are mainly the C-H···π and C-H···B systems.

## 3. The Case of Halogen Bonds

There are several various interactions that are treated in numerous studies as the hydrogen bond counterparts, particularly it concerns so-called σ-hole and π-hole bonds [38,39,40,41,42]. The σ-hole is a region of electron charge depletion at a centre considered approximately in the direction from an atom bonded to this centre, in the elongation of this bond [38,39]. On the other hand, the π-hole concerns a centre in a planar molecule or a planar molecular fragment [41,42], the triel centres such as boron one in trihalides and trihydrides are examples of such a situation [43,44]. The electron charge depletion at the σ-holes and π-holes often leads to the positive electrostatic potential, EP, at these regions; hence they often act as the Lewis acid centres.

**Figure 3:**
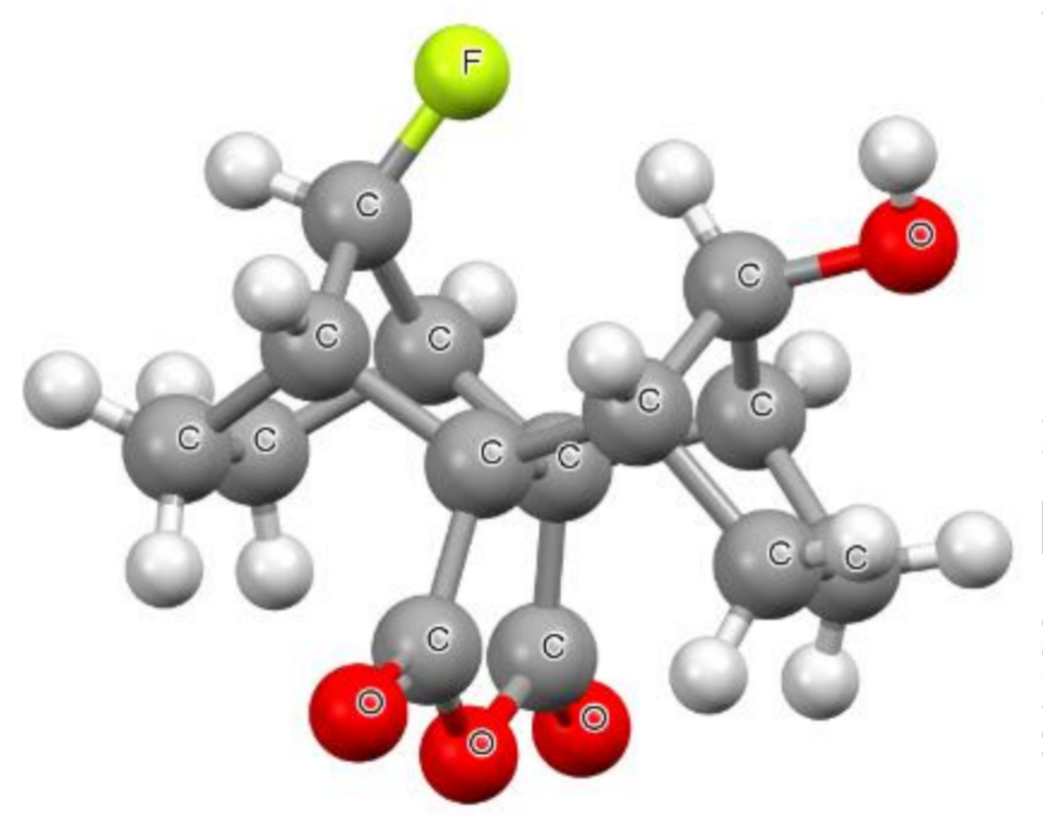
The fluoronium ion precursor from the crystal structure [50], BEXNOJ refcode.

The halogen bond, XB, is a case of an interaction where the σ-hole at halogen centre, X (F, Cl, Br, I or At) i.e., the Lewis acid site, interacts with the Lewis base centre [38,39]. This is why this interaction is often considered as the hydrogen bond counterpart, in numerous studies the comparison of these interactions is performed [11,40,45,46,47]. However, there is the question if XB, similarly as the hydrogen bond, may be considered as a preliminary stage of the chemical reaction. The PT process follows sometimes the hydrogen bond formation. Does a similar transfer of a halogen atom occur? Let us discuss shortly studies, particularly the recent ones, on the entities with halonium cation that links two Lewis base centres. The latter topic is revealed in recent studies by two challenges. The first one concerns finding of Lewis base–halonium ion–Lewis base arrangements with asymmetrically located halonium cation [48]. They are designated later here as [LbXLb]^+^, where in further discussions Lb is replaced by the specific atomic centre while X marks the halogen. The second challenge concerns finding of the [LbXLb]^+^ arrangement with fluorine, X = F, situated between the Lewis base sides since rather such systems with heavier halogens are known [49]. As concerns the latter challenge, the generation of a symmetrical fluoronium ion in solution was discussed, its existence was evidenced indirectly [50]. These experimental studies were supported by the calculations performed at various levels, all levels applied confirmed this ion existence, for example, the B3LYP/6-311++G(d,p) results show equal F···C distances amounting 1.6 Å in the [CFC]^+^ arrangement [50]. It was proved in numerous earlier and recent studies that the heavier halogen atom (Cl, Br, I) may be engaged in the hypervalent link in solution possessing formally positive charge. Such a link was usually doubted for the fluorine centre; the studies of Lectka and co-workers discussed here [49,50] are related to this challenge. However, it was evidenced only indirectly the occurrence of the fluoronium ion as a short-lived reaction intermediate [50]. It was discussed also that this ion is formed in a solvolysis process from a precursor molecule probably according to the S_N_1 reaction through the fluorine centre [50,51]. The crystal structure of the fluoronium ion precursor was determined [50]; the structure of this ion taken from the Cambridge Structural Database, CSD [17,18] is presented in Figure 3. The direct observation of the similar symmetrical [CFC]^+^ ion was described recently [49], this is a stable species in solution and it was characterised by ^19^F, ^1^H, and ^13^C NMR.

**Figure 4:**
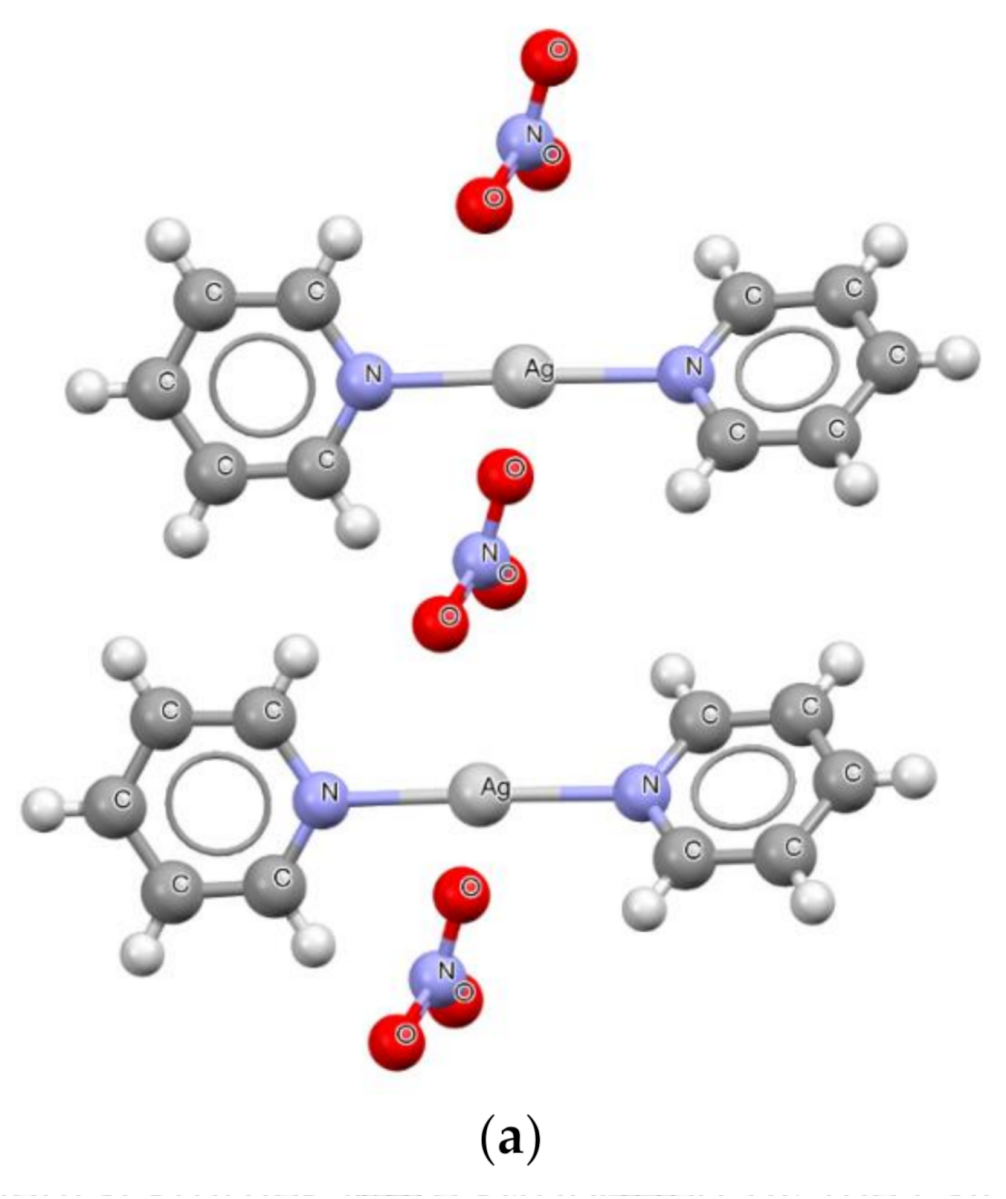

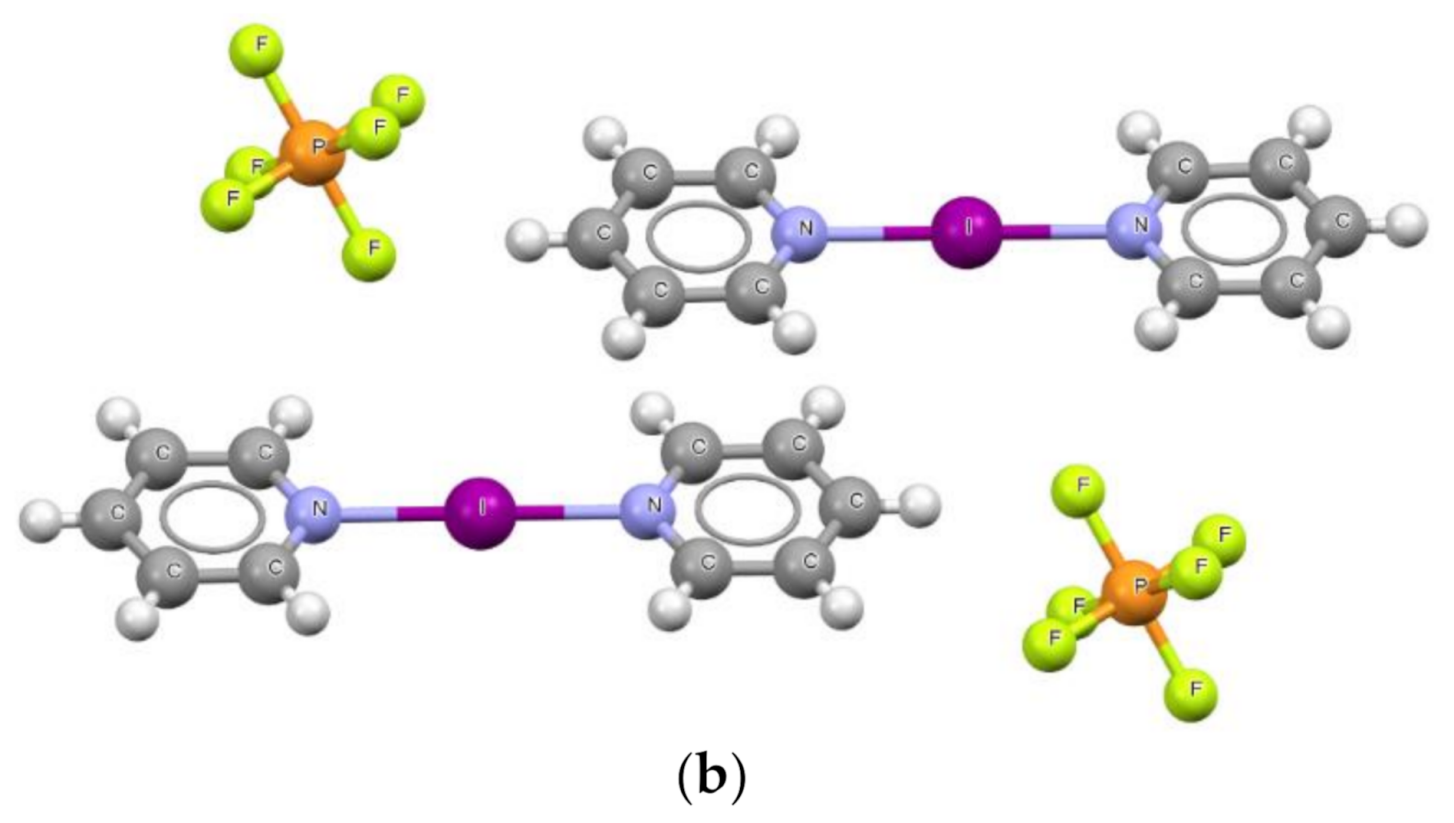
The fragment of the crystal structure of bis(pyridine)-silver(i) nitrate monohydrate, AGPYNO02 refcode (**a**) and di(pyridin-1-yl)iodonium hexafluorophosphate, CICQIQ03 refcode (**b**), reference [53].

**Figure 5:**
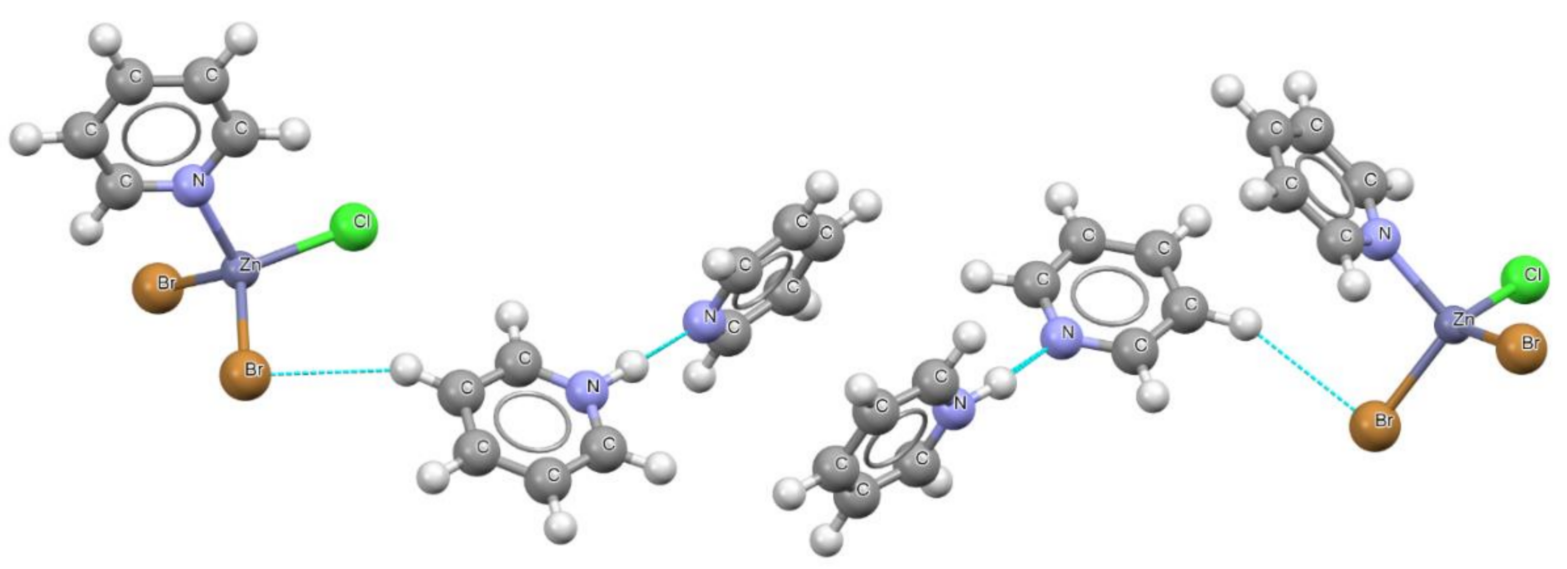
The fragment of the crystal structure of pyridinium dibromo-chloro-pyridine-zinc(ii) pyridine solvate, PYCBZN01 refcode, reference [54].

Another challenge announced here concerns asymmetric [NXN]^+^ systems since the symmetrical systems containing the halogen, X, are known rather from various studies [48]. For example, the experimental NMR spectroscopic and crystal structure studies as well as the theoretical calculations were performed recently on the systems containing halogen cation as well as other cations between nitrogen centres, i.e., the [NZN]^+^ systems where Z^+^ = H^+^, Li^+^, Na^+^, F^+^, Cl^+^, Br^+^, I^+^, Ag^+^ and Au^+^ [52]. Two series, one of bis(pyridine) entities and the second one containing the (1,2-bis(pyridin-2-ylethynyl)benzene) structure were considered [52]. In all cases the symmetrical arrangements are observed, only for Z^+^ = H^+^ and F^+^ the asymmetric systems occur; this concerns both above-mentioned series. However, in a case of the fluorine species there is no experimental confirmation of such asymmetry, only theoretical calculations reveal such arrangements. Figure 4 and Figure 5 show fragments of bis(pyridine) crystal structures. In two cases (Figure 4); silver [53] and iodine [53] cations are located in the centre of the [NAgN]^+^ and [NIN]^+^ systems, respectively. In a case of the bis(pyridine)proton structure the asymmetric [NHN]^+^ arrangements are observed [54] (see Figure 5).

It is worth mentioning that the potential energy curves were analysed theoretically for the bis(pyridine) series of complexes [52], the displacement of the Z^+^ cation from the N···N mid-point was plotted versus the electronic energy of the system. The single well potential energy curve is observed for all systems except of Z^+^ = H^+^, F^+^ where the double minimum symmetrical curve occurs [52]. Since the similar situation occurs for the series of 1,2-bis(pyridin-2-ylethynyl)benzene structures thus one can expect that the potential energy curve shape depends on the cation. However, this shape is a result of numerous factors; not only a kind of a cation. It depends on the type of a complex, the environment that results from the arrangement of molecules in crystals or from the type of solvent in liquids. For example, the first asymmetric linear silver complexes and the first asymmetric halonium complexes characterised by the [NAgN]^+^ and [NIN]^+^ arrangements, respectively, were analysed and they were confirmed by ^1^H and ^1^H-^15^N HMBC NMR spectroscopy, and by X-ray diffraction results in crystals [55]. Thus one may expect that the halonium ion transfer and the transfer of other cations as Ag^+^, for example, is possible for some systems. It is worth to mention that Zundel compared the hydrogen bond with analogues interactions where the proton is replaced by the lithium, sodium or potassium cations; the transfers similar to the proton transfer were analysed [56].

Let us compare systems containing Z^+^ cations that were discussed above with the hydrogen bonds. The examples presented earlier here indicate the asymmetric position of proton in [NHN]^+^ arrangement for both 1,2-bis(pyridin-2-ylethynyl)benzene and bis(pyridine) structures. It seems that the asymmetry of the H-atom position results, at least partly, from the asymmetry of environment in crystals. In another crystal structure containing protonated homodimer of pyridine, i.e., pyridinium tetrakis(3,5-bis(trifluoromethyl)phenyl)borate pyridine solvate, the asymmetry of the [NHN]^+^ arrangement is also observed; however the asymmetric [NHN]^+^ bridges occur also in solution [57]. The NMR signals in solution show the fast reversible proton transfer. All results concerning crystal as well as solution indicate the asymmetry of the [NHN]^+^ contact, in spite the N-H∙∙∙N hydrogen bond is very strong and covalent in nature and the weak anion coordination is observed. Other strong N-H∙∙∙N hydrogen bonds in derivatives of proton-bound homodimer of pyridine were analysed in solution and it was also found they are asymmetric in spite of these being very strong interactions [58].

It is worth to note however that hydrogen bonds characterised by a single symmetrical minimum of potential energy curve are also well known. The [FHF]^−^ anion is an example, the symmetrical species is known in the gas phase [59] although in crystal structure this anion is disturbed by packing forces since the movement of proton from the central position is often observed [60]. Similarly, the OH···O hydrogen bonds were analysed as it was discussed which conditions have to be fulfilled for the central position of proton [61]. Figure 1 presented earlier here shows geometries of numerous OH···O systems, the asymmetric ones as well as those where the proton is very close to the O···O mid-point. Various studies on hydrogen bonds, and particularly on the O-H···O systems indicate the complex character of PT process [62], for example, the potential barrier height should be taken into account in the analysis of this process. The same probably concerns the transfer of other cations, among them of halonium ones. There is no sufficient experimental and theoretical results however, and there is room for more extended investigations in this matter.

## 4. The Dihydrogen Bond as a Stage of the Molecular Hydrogen Uptake

The dihydrogen bond, DHB, is a special type of the hydrogen bond where the Lewis base centre is the negatively charged hydrogen atom [63,64,65]. In other words, it is the link between hydrogen atoms of the opposite charge, H^+δ^···^−δ^H. The question arises if the proton transfer process A-H∙∙∙B ⇔ A^−^∙∙∙^+^H-B known for the A-H∙∙∙B hydrogen bonded systems [62] occurs for dihydrogen bonds. This is discussed in detail in the monograph of Bakhmutov where numerous studies related to this topic are discussed [65]. It was generalised that the protonation of the hydride species characterised by the negatively charged hydrogen centre may be reversible or not, the latter phenomenon is connected with the molecular hydrogen elimination that may be expressed by the following transformations.

(2) $$A-H^{+\delta }+{}^{-\delta }H-B\;\to \;{\left[B\left(H_{2}\right)\right]}^{+}A^{-}\;\to \;A^{-}+H_{2}+B^{+}$$

It is worth mentioning that the B centre connected with the hydric hydrogen is most often the transition metal, the numerous moieties containing molecular hydrogen attached to the transition metal centre are known, such systems often occur in crystal structures [66,67]. The model F-H^+δ^∙∙∙^−δ^H-Li complex corresponding to the phenomena expressed by Equation (2) was analysed theoretically early at the HF/6-31G(d,p) level [68]. It was found that the presence of the external electric field may lead to the formation and elimination of the molecular hydrogen since there are the following products; F^−^ + H_2_ + Li^+^, of the proton transfer reaction. The reverse process is observed for the system containing dihydrogen molecule inserted between Lewis acid and Lewis base units, H_3_B∙∙∙H_2_∙∙∙NH_3_ since the DHB system is formed in the external electric field; H_3_B^−^-H∙∙∙H-^+^NH_3_ [68]. Bakhmutov, in his monograph [65], gives numerous examples of PT process in DHBs systems. The PT reaction and the dihydrogen elimination were discussed in the X-ray crystal structure of *N*-[2-(6-aminopyridyl)]acetamidine cyanoborohydride [69], the solid state transformation from the dihydrogen bonded LiBH_4_-triethanolamine system to the covalent bonded material was described in another study [70].

There is an early example of the theoretical study [71] where the following reaction that leads to the molecular hydrogen uptake through the dihydrogen bond formation and next the proton transfer was analysed for a series of complexes of the AlH_4_^−^ anion.

(3) $${\left[{\mathrm{AlH}}_{4}\right]}^{-}+{\mathrm{HX}\;\to \;\mathrm{AlH}}_{3}-H^{-}\cdots \mathrm{HX}\;\to \;{\left[{\mathrm{AlH}}_{3}X\right]}^{-}+H_{2}$$

The MP2/6-311++G(d,p) calculations were performed for this process (Equation (3)) with the HF, HCl, and H_2_O species acting as the proton donors. The transition states of the process of transformation from the DHB systems to the products containing dihydrogen molecule were also calculated indicating that the PT reactions are energetically possible here, for example the potential barrier height for the AlH_3_-H^−^∙∙∙HCl → [AlH_3_Cl]^−^ + H_2_ reaction is equal to 6.7 kcal/mol.

In one of more recent studies [72] the reverse process of the molecular hydrogen cleavage at the metal centre that leads to the dihydrogen bonded complex formation was analysed; FM + H_2_ → FH∙∙∙HM (M = Cu, Ag, Au). The calculations were performed with the use of the aug-cc-pVQZ basis set (aug-cc-pVQZ-PP for metal atoms) and the following methods, MP2, CCSD(T) and CASSCF/CASPT2. The systems with dihydrogen molecule attached to the metal centre correspond to global minima, while the dihydrogen bonded complexes are characterised by higher energies (local energetic minima). Only for complexes containing the gold centre the DHB system is lower in energy than the system with the H_2_ molecule for CASSCF/CASPT2 and CCSD(T) calculations. For example, for the CCSD(T)/aug-cc-pVQZ(aug-cc-pVQZ-PP) level of calculations for the FAu + H_2_ → FH∙∙∙HAu reaction the potential barrier height amounts 30.9 kcal/mol and the products of reaction are lower in energy than reactants by 1.5 kcal/mol.

## 5. The Change of Trigonal Planar Triel Configuration into the Tetrahedral One—Triel Bonds

The triel bond is an interaction between the 13th group element acting as the Lewis acid centre and the electron-rich region of another or the same species [43,44,73]. It is worth to note that the acidic properties of triel atoms are related to the so-called π-holes [41,42] that are regions in planar molecules or in planar fragments of molecules at centres characterised by the depletion of the electron charge. For example, the trivalent triel atoms in trihydrides and trihalides possess formally empty p-orbital situated perpendicularly to the planes of molecules and being capable to act as the electron acceptor; numerous early [74,75,76,77] and more recent studies [43,44,73] related to interactions of the above-mentioned species with Lewis base units are known. The special attention should be paid to the studies of Phillips and co-workers who analysed such complexes from the theoretical and experimental points of view since the high level ab initio calculations were performed as well as the corresponding crystal structures were discussed [78,79,80,81,82]; particularly the potential energy as a function of the distance between the Lewis acid-base units was described [78]. It was found that the double local minimum occurs for some of complexes, deeper one corresponding to shorter distances and the flat, shallow energy minimum for the longer distances. The former minimum corresponds to strong and partly covalent interactions while the latter local minimum to weak interactions characterised mostly by dispersion forces. These findings are in line with other more recent studies [73,83].

It is worth to mention that for the triel bonds, similarly as for the hydrogen bonds, different sub-classes of interactions exist [84]. There are triel bonds with the one-centre electron donor, A-T…B (where T is the triel atom, B is the electron donor and A is any atom connected with the triel centre), the interactions with π-electrons acting as the electron donor, A-T∙∙∙π where the alkenes and alkines were considered as the Lewis base units [85,86] as well as benzene entity [87]. Another sub-class concerns links where the σ-bonds´ electrons play a role of the electron donors, A-T∙∙∙σ interactions; the complexes of molecular hydrogen acting as the Lewis base unit with the boron trihydride and boron trihalides were analysed in early study [85] and more recently the complexes of molecular hydrogen with boron and aluminium trihydrides and trifluorides were analysed theoretically [88]. Finally, one can mention intramolecular and bifurcated triel bonds that occur in the crystal structures [89]. It is well known that such types of hydrogen bonds are common in crystals [1,2,90].

**Scheme 1:**
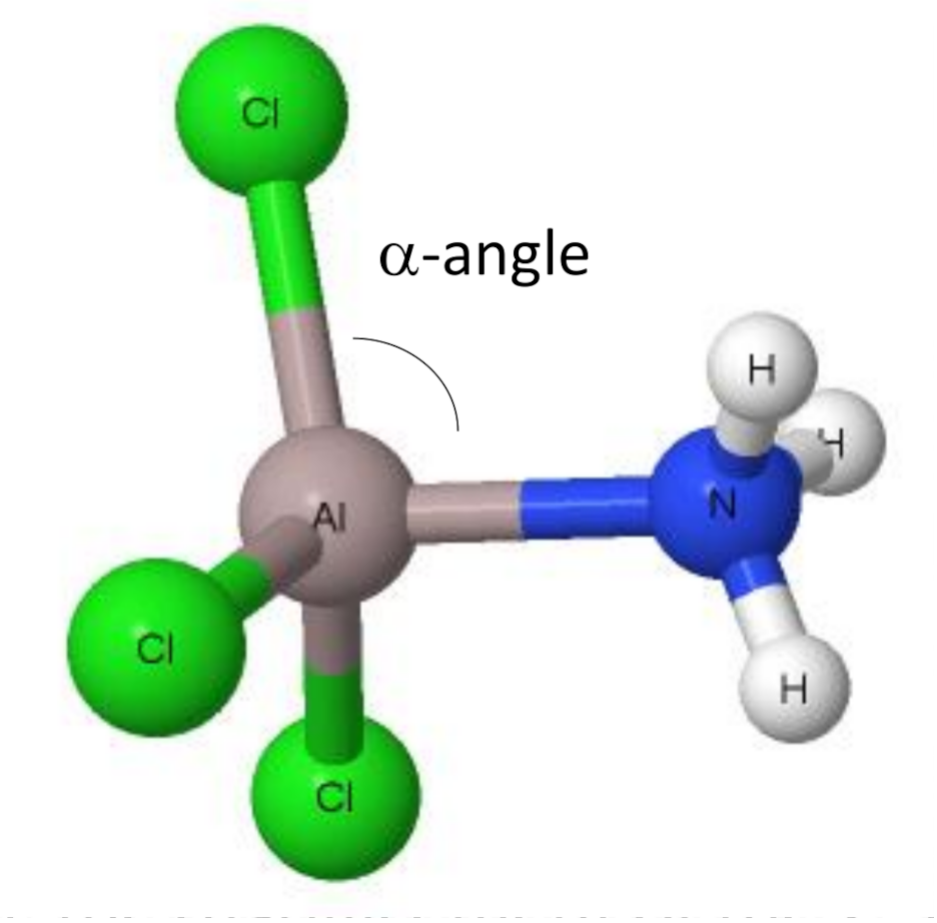
The AlCl_3_∙∙∙NH_3_ complex linked by the Al∙∙∙N triel bond.

The most interesting is which structural changes follow the formation of triel bonds? For example, the trivalent triel species described shortly above here change their planar structure if they interact with Lewis bases. The stronger interactions result in greater changes in the triel species geometry; in a case of extremely strong interactions, for example with anions, the triel centre conformation becomes to be close to tetrahedral structures. Even it acquires an ideal T_d_ symmetry like in the BF_4_^−^ anions which occur very often in crystal structures [91]. Scheme 1 presents an example of the AlCl_3_∙∙∙NH_3_ complex linked by the Al∙∙∙N triel bond. The α-angle which may be treated as the parameter of the deformation resulting from complexation is defined in this scheme. It is the angle between the Al∙∙∙N line corresponding to the triel bond and one of the Al-Cl bonds. The angle parameter may be applied to other simple triel species complexes that informs the Lewis acid unit deformation resulting from the triel bond formation. This angle is equal to 90° if the interaction is very weak and the molecular deformations are not detected, it means that the triel Lewis acid species is still planar, or at least it is close to planarity. The increase of the α-angle is observed with the increase of the strength of interaction, for the BF_4_^−^ anion being the result of the BF_3_…F^−^ interaction it amounts 109.5° like for methane and other species possessing T_d_ symmetry.

**Figure 6:**
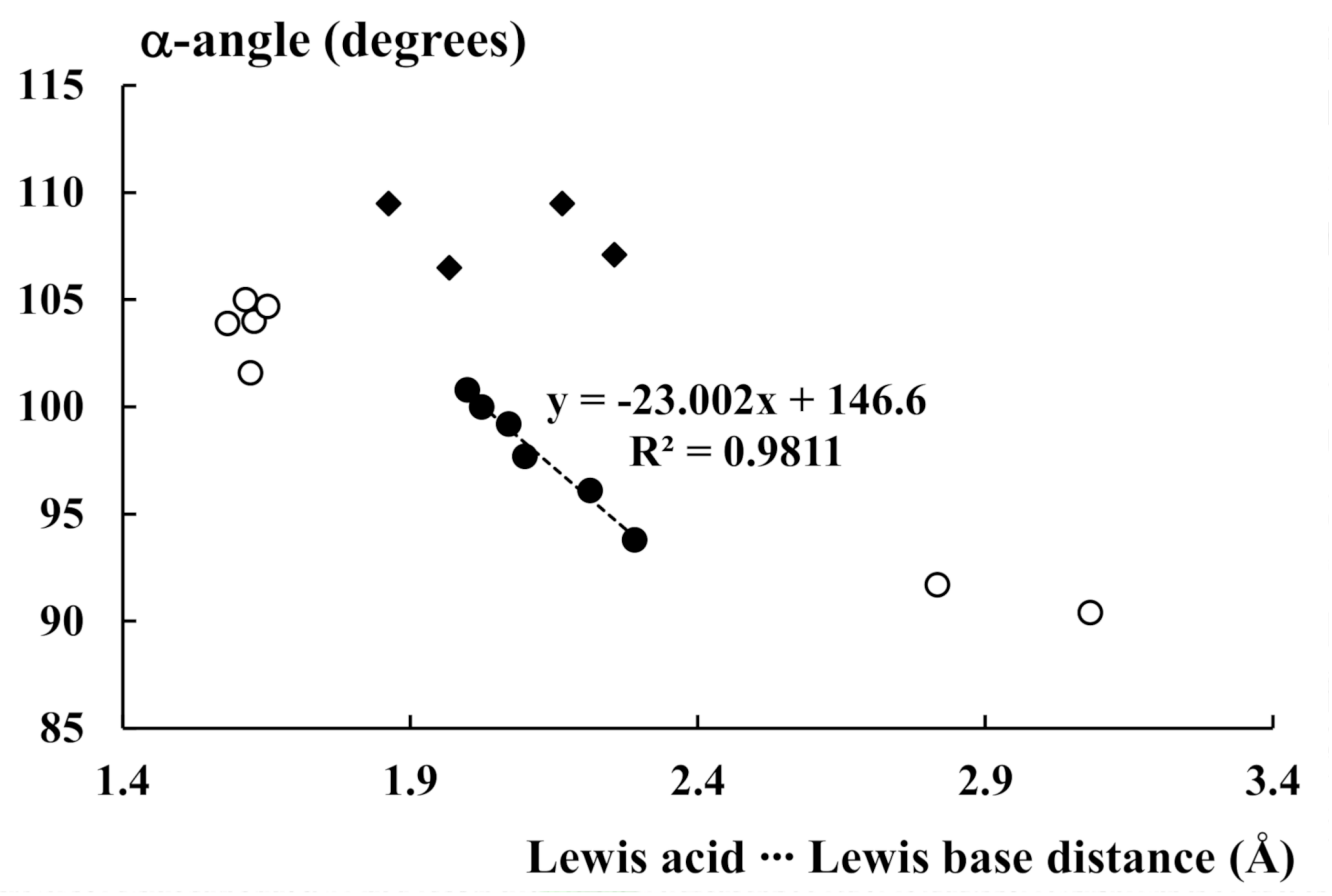
The dependence between the Lewis acid∙∙∙Lewis base distance, T∙∙∙B, (Å) and the α-parameter (see Scheme 1) in A-T∙∙∙B triel bonds (T-triel centre). Black and white circles correspond to the neutral complexes, aluminium and boron species, respectively. Squares correspond to anion complexes.

Figure 6 presents the relationship between the triel centre - Lewis base centre distance and the α-angle for simple complexes of boron and aluminium trihydrides and trichlorides. This dependence is based on the MP2/aug-cc-pVTZ results that come from one of recent studies [73]. The following Lewis base units were taken into account there; HCN, NH_3_, N_2_ and Cl^−^. In such a way three sub-classes of complexes are presented in Figure 6; the ionic species being complexes with chloride anion and the remaining complexes are divided into two groups: complexes of aluminium Lewis acid units and complexes of boron compounds. The excellent linear correlation is observed for the neutral complexes of aluminium. For the remaining complexes, only tendency is observed that for stronger interactions (shorter Lewis acid-base distances) the greater α-angle occurs. In two cases of ionic systems, BCl_4_^−^ and AlCl_4_^−^ the ideal T_d_ symmetry (α-angle equal to 109.5°) is observed certainly.

Thus one can see that the relationship presented in Figure 6 may be treated as the reaction path for the process of the change of trigonal and planar configuration into the tetrahedral structure; few complexes linked by the triel bond are stable structures characterised by large dissociation energies. It is interesting that these boron adducts characterised by the structures close to tetrahedral ones may further interact with the electron rich species according to the S_N_2 reaction mechanism. The corresponding studies related to this topic appear from time to time, for example the reactions of borine carbonyl with trimethylamine and triethylamine were analysed experimentally early [92,93], or in more recent study, the S_N_2 reaction for the Cl^−^∙∙∙CH_3_Cl and NH_3_∙∙∙H_3_BNH_3_ complexes were analysed theoretically [94], the latter one concerns the tetrahedral structure of boron.

## 6. Tetrel Bonds and the S_N_2 Reaction

**Scheme 2:**
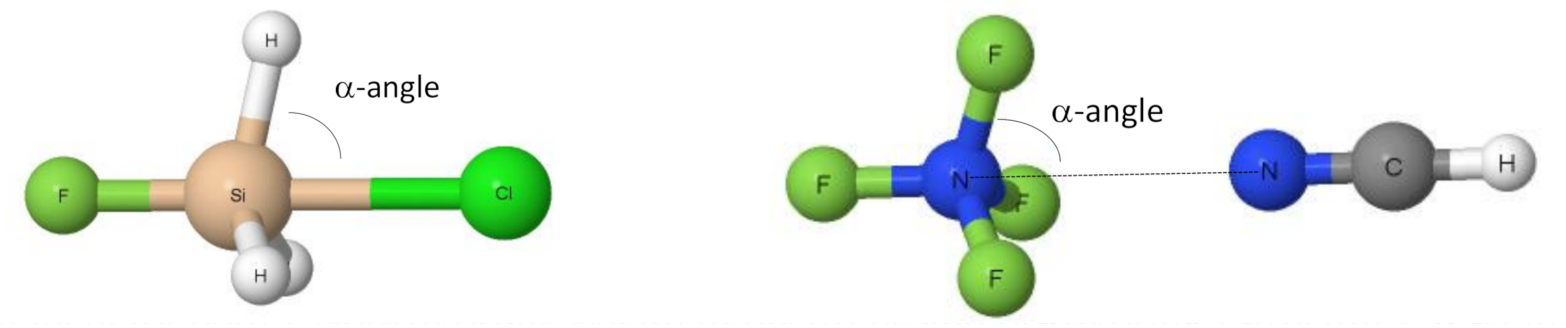
The SiFH_3_∙∙∙Cl^−^ and NF_4_^+^∙∙∙NCH complexes linked by the tetrel bond and pnicogen bond, respectively.

The tetrel bond is an interaction between the tetrel centre (14th group element) of the Lewis acid unit and the electron rich region of the moiety playing a role of the Lewis base [41,42,95,96,97,98]. The tetrel centre region that is responsible for the acidic (electron accepting) properties is classified as the σ-hole [95] but there are also cases of the tetrel centres that possess π-hole regions [98]. If the tetrel centre interacts with the Lewis base through the σ-hole thus the corresponding tetrel bond is often a preliminary stage of the S_N_2 reaction [97]. The tetrel bond as a type of the σ-hole bond leads to the geometrical changes of interacting units that the complex structure is close to the trigonal bipyramid, especially in a case of strong interactions [97]. Scheme 2 presents an example of the species connected by the σ-hole tetrel bond. It is the SiFH_3_∙∙∙Cl^−^ complex with the Si∙∙∙Cl intermolecular link and the SiH_3_ group in the central part of the complex that tends to the planarity. The latter situation is similar to those observed for transition states of S_N_2 reactions. Hence the similar parameter to that one discussed earlier here for the triel bonds (Scheme 1) may be introduced for tetrel σ-hole bonds. It is the α-angle (Scheme 2) between the tetrel∙∙∙Lewis base link and one of bonds of the tetrel species. For the example shown in Scheme 2 it is the angle between Si∙∙∙Cl line and the Si-H bond. For very strong interactions this angle is equal to 90° or nearly so. On the contrary, for extremely weak interactions that do not disturb the geometry of the Lewis acid unit the α-angle should be equal to ~70.5° that corresponds to the ideal tetrahedral structure of the tetrel species that is not perturbed by external forces.

This is interesting that similar characteristics are often observed for the charge assisted pnicogen species, which interact with the Lewis base units [99]. The complexes of ZH_4_^+^, ZF_4_^+^ and ZFH_3_^+^ cations (Z = N, P, As) were analysed theoretically and the Z∙∙∙Lewis base intermolecular links were observed [99] that may be classified as the type of σ-hole bonds, i.e., pnicogen bonds. These charge assisted pnicogen bonds also lead to the structural changes similar to those occurring for tetrel bonds, and they may be also classified as the preliminary stages of the S_N_2 reactions [99]. Scheme 2 presents the NF_4_^+^∙∙∙NCH complex connected by the N∙∙∙N pnicogen bond. One of the nitrogen centres plays here a role of the electron acceptor while another N-centre is the electron donor. This is a very similar situation to that one occurring for the σ-hole tetrel bonds discussed here (see the same Scheme 2 with an example of the tetrel bonded complex).

**Figure 7:**
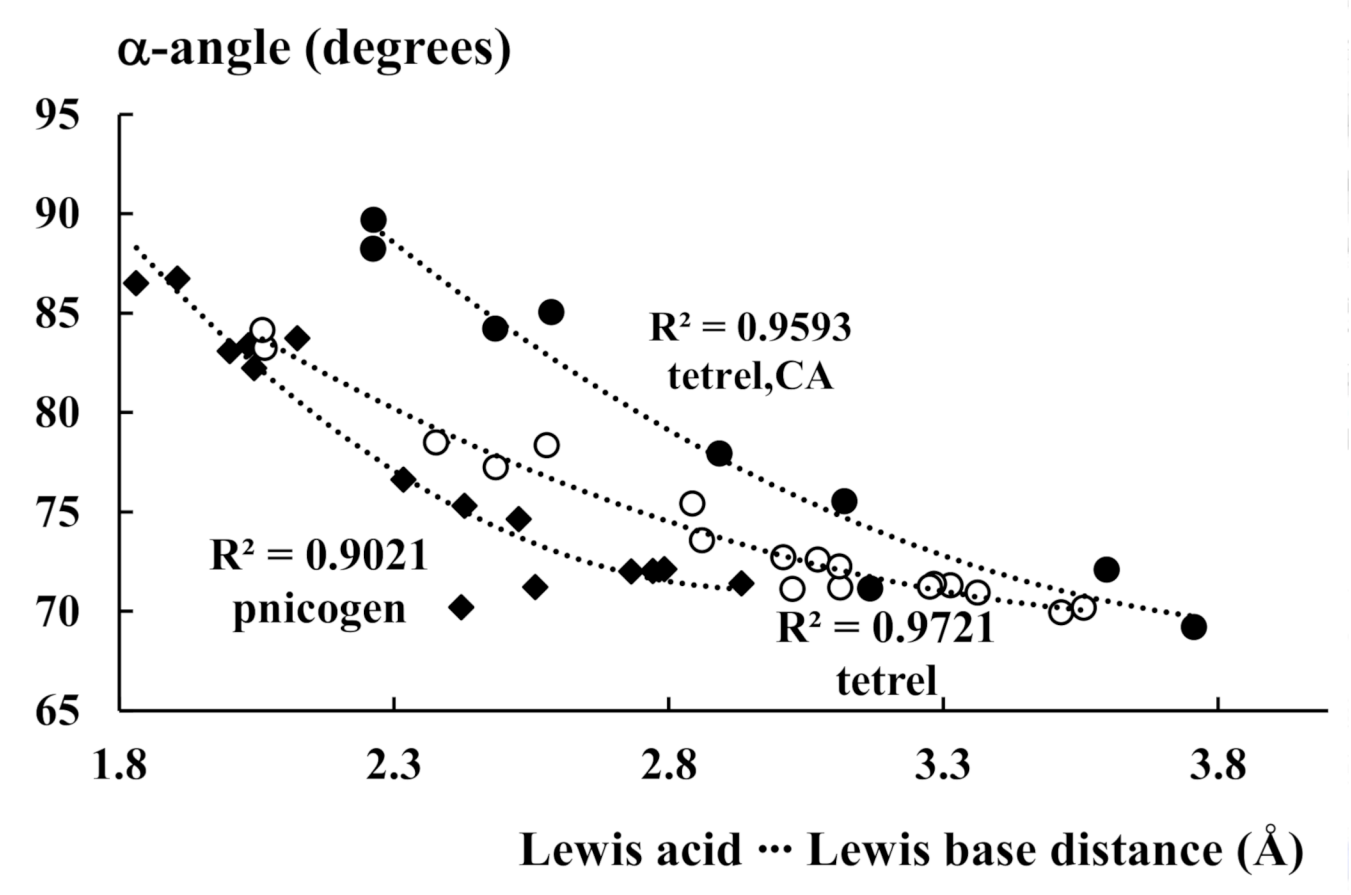
The dependence between the Lewis acid∙∙∙Lewis base distance, Z∙∙∙B, (Å) and the α-parameter (see Scheme 2) in A-Z∙∙∙B tetrel and pnicogen bonds (Z-tetrel or pnicogen centre). Black and white circles correspond to the tetrel complexes linked by charge assisted tetrel bonds and neutral tetrel bonds, respectively. Squares correspond to complexes linked by the pnicogen bond.

Figure 7 presents the relationships between the tetrel/pnicogen centre∙∙∙Lewis base centre distance and the α-angle. Two sub-samples of complexes of tetrel species and one sub-sample of pnicogen species complexes are presented here. These geometrical results were taken from earlier studies [97,99] where the MP2/aug-cc-pVTZ calculations were performed on complexes presented in this figure. Let us describe the sub-samples presented in Figure 7. For that one with units linked by tetrel bond, the ZH_4_, ZFH_3_ and ZF_4_ (Z = C, Si, Ge) species interact with the HCN and LiCN acting as Lewis bases (nitrogen atom is the Lewis base centre here). For the complexes with negatively charge assisted tetrel bonds the same Lewis acid units as for the former sub-sample interact with chloride anion. And for complexes linked by the pnicogen bond the ZH_4_^+^, ZFH_3_^+^ and ZF_4_^+^ species (Z = N, P and As) interact with HCN and LiCN through the Z and nitrogen centres.

Figure 7 shows that for shorter Lewis acid-base distances corresponding to stronger interactions, the α-parameter (Scheme 2) tends to 90°. It means that the central part of the complex becomes to be planar as it is observed for the transition state of the S_N_2 reaction. The second order polynomial dependences between the distance and the α-angle are observed here with high values of the correlation coefficients. These dependencies may be treated as the corresponding reaction paths of the above-mentioned S_N_2 reactions. This is very interesting that the pnicogen and tetrel σ-bonds considered here (Figure 7) do not practically differ between themselves. For both interactions the Lewis acid units possess the tetrahedral structure, for both interactions, the complexation leads to the geometrical changes towards the structure of trigonal bipyramid. And finally, both interactions may be considered as the preliminary stages of the S_N_2 reactions.

One can also mention C-H∙∙∙M (M designates metal) contacts that often play an important role in catalysis [100]. These interactions are usually classified as attractive ones and they are named as agostic interactions in terms of the Dewar-Chatt-Duncanson model [101,102]. However, the term anagostic interactions was introduced [103] for such contacts that are sterically enforced in square-planar transition metal d^8^ complexes to distinguish them from attractive agostic interactions. The orbital interaction schemes were presented by Scherer and co-workers for various types of the C-H∙∙∙M interactions [104]; these are: the above-mentioned repulsive anagostic 3c-4e interaction being the contact between hydric hydrogen and the filled M-dz^2^ orbital, the attractive 3c-4e hydrogen bond being the interaction between the protic H-atom and the filled M-dz^2^ orbital, preagostic attractive 3c-2e interaction (π-back donation) and the σ-agostic 3c-2e attractive interaction (hydric hydrogen–empty M-dz^2^ orbital). On the other hand, in another study it was justified that the formation of numerous C-H…M structures is mainly driven by the dispersion forces thus the models based on the orbital-orbital interactions schemes are not sufficient to describe the nature of these structures [105]. It seems that both explanations, the “orbital based” explanation as well as that one considering dispersion forces, are valid. For example, in one of recent studies on the C-H…Ni contacts in Ni^II^ planar isomers the occurrence of the covalent type charge delocalisation and of the London dispersion forces was justified [106]. The latter study is based on experimental results that are supported by theoretical analyses where various approaches were applied [106]. However, it is worth noting that these C-H…M interactions, regardless of the mechanism of their formation, lead to the metal centre coordination change. In the case of square-planar structures with two additional C-H…M contacts the metal structure tends to the octahedral one. Hence the agostic interaction may be considered as a preliminary stage of the process of structural reconstruction.

## 7. Summary

Different interactions are discussed in this article; the hydrogen bond and its special type—the dihydrogen bond, the pnicogen, and tetrel bonds as representatives of the σ-hole bond, and the triel bond as an example of the π-hole bond. These interactions may be treated as preliminary stages of various reactions and processes; the proton transfer, the release of the molecular hydrogen, or the S_N_2 reaction.

However, it is also very important that intra- and intermolecular connections lead to numerous structural changes of the interacting units. The triel planar and trigonal species interacting with Lewis bases tend to achieve the tetrahedral geometry. On the other hand the tetrahedral tetrel moieties as well as the tetrahedral pnicogen cations, both characterised by the lack of the lone electron pairs, tend to attain the structure of the trigonal bipyramid. It is worth to mention that different structural changes take place for different species of the same element, like in a case of the σ-hole bonds on one hand and in a case of the π-hole bonds on the other hand. In general, there is a variety of numerous structural changes accompanying the processes of complexation.

Finally it is worth noting here that other interactions not discussed here may initiate various chemical reactions, there is room for numerous future studies related to this topic. One of the most important topics to be discussed in next studies is the application of the structure-correlation method [12,13,14]. This method may be generalised as the analysis of geometrical changes during the chemical reactions. One can see that Figure 1 and Figure 2 presented in this study express the changes following the proton transfer process, Figure 6 shows such changes related to the transformation of the trigonal planar system into the tetrahedral structure while Figure 7 shows the reaction paths of the S_N_2 reactions.
